# Oxidative Stress and Inflammatory Mediators in Exhaled Breath Condensate of Patients with Pulmonary Tuberculosis. A Pilot Study with a Biomarker Perspective

**DOI:** 10.3390/antiox10101572

**Published:** 2021-10-05

**Authors:** Silvia Guzmán-Beltrán, Laura Elena Carreto-Binaghi, Claudia Carranza, Martha Torres, Yolanda Gonzalez, Marcela Muñoz-Torrico, Esmeralda Juárez

**Affiliations:** 1 Departamento de Investigación en Microbiología, Instituto Nacional de Enfermedades Respiratorias Ismael Cosío Villegas, Calzada de Tlalpan 4502, Sección XVI, Tlalpan, Ciudad de México 14080, Mexico; sguzman@iner.gob.mx (S.G.-B.); ygonzalezh@iner.gob.mx (Y.G.); 2 Laboratorio de Inmunobiología de la Tuberculosis, Instituto Nacional de Enfermedades Respiratorias Ismael Cosío Villegas, Calzada de Tlalpan 4502, Sección XVI, Tlalpan, Ciudad de México 14080, Mexico; lecarreto@iner.gob.mx (L.E.C.-B.); claudia.carranza@iner.gob.mx (C.C.); mtorres@iner.gob.mx (M.T.); 3 Servicio Clínico de Tuberculosis, Instituto Nacional de Enfermedades Respiratorias Ismael Cosío Villegas, Calzada de Tlalpan 4502, Sección XVI, Tlalpan, Ciudad de México 14080, Mexico; dra_munoz@iner.gob.mx

**Keywords:** exhaled breath condensate, inflammation, oxidative stress, pulmonary tuberculosis

## Abstract

Tuberculosis (TB) is one of the highest infectious burdens worldwide. An excess of inflammation and inadequate antioxidant defense mechanisms are believed to lead to chronic inflammation and lung damage in tuberculosis (TB). However, circulating metabolites do not always replicate lung-associated biomarkers that define the pathobiology of the disease. The objective of this study was to determine the utility of exhaled breath condensate (EBC), a non-invasive and straightforward sample, to evaluate alveolar space-derived metabolites of redox state and inflammation. We assessed the levels of exhaled oxidant/antioxidant parameters (8-isoprostane, MDA, GSH), inflammatory markers, such as nucleosomes, cytokines (IL-2, IL-4, IL-6 and IL-8, IL-10, GM-CSF, TNF-α, and IFN-γ) and lipid mediators (PGE2, LTB4, RvD1, and Mar1), in patients with recently diagnosed pulmonary TB and healthy controls’ EBC and serum. The TB patients showed 36% lower GSH levels, and 2-, 1.4-, 1.1-, and 50-fold higher levels of 8-isoprostanes, nucleosomes, IL-6, and LTB4, respectively, in EBC. There was no correlation between EBC and serum, highlighting the importance of measuring local biomarkers. Quantitation of local inflammatory molecules and redox states in EBC would help find biomarkers useful for pharmacological and follow-up studies in pulmonary tuberculosis.

## 1. Introduction

Tuberculosis (TB) is one of the highest infectious burdens worldwide (Global TB report 2020). TB patients who are successfully treated are at high risk of residual pulmonary dysfunction. Pulmonary fibrosis and dysfunction in TB result from chronic inflammation in the lung, and inflammatory mediators such as oxygen radicals, cytokines, and eicosanoids released by immune and nonimmune cells are critical for sustaining inflammation [1,2,3,4,5].

Constant oxidative stress may contribute to the development of lung dysfunction because patients with pulmonary TB have increased circulating markers of free radical activity, some of which remain elevated even after the conclusion of antimycobacterial chemotherapy [6]. Patients with TB have elevated plasma levels of oxidative stress markers such as malondialdehyde (MDA) and conjugated dienes and decreased antioxidant effectors such as superoxide dismutase (SOD), catalase (CAT), and glutathione peroxidase (GPx) and glutathione (GSH) [7].

The cytokines play an essential role in the regulation of inflammatory responses. In the early stages of infection, antigen-presenting cells (APCs) release IL-6 and IL-12, followed by the synthesis of IFN-α, IL-23, IL-1β, and IL-17 by lymphocytes [8,9,10], which further activate macrophages to produce TNF-α [11]. Subsequently, TNF-α promotes the intracellular elimination of the mycobacteria by producing reactive oxygen and nitrogen species (ROS and RNS) and contains the pathogen by contributing to granuloma maintenance [11,12,13]. However, the hyperinflammation often observed in TB causes lung tissue necrosis and cavitation, facilitating TB transmission [14,15].

Eicosanoids provide balance in the inflammation in TB; whereas the proinflammatory lipids activate at the beginning of the infection, the pro-resolving members of the eicosanoid family are responsible for the resolution [16]. Lipid mediators are hormone-like substances that modulate the outcomes of experimental M. tuberculosis infection. Leukotriene B4 (LTB4) and prostaglandin E2 (PGE2) play a host protective role by mediating bacterial clearance, while lipoxin A4 (LXA4) and 15-epi-lipoxin A4 (15-epi-LXA4) play a pathogenic role by hampering the host inflammatory response in TB [3,17]. Virulent strains of M. tuberculosis induce the production of LXA4, which inhibits the production of PGE2, while non-virulent strains preferentially induce the expression of PGE2. PGE2 is critical in polarizing immune responses towards a Th1-type response and preventing plasma membrane damage. Thus, the balance between pro and anti-inflammatory eicosanoids is critical for the modulation of the host response to M. tuberculosis infection and the control of TB immunopathology [17,18].

The inflammatory and redox status of patients with pulmonary TB is commonly inferred by analyzing plasma or serum metabolites, where the evaluated parameters rarely represent the lung microenvironment. The bronchioloalveolar lavage is the best sample to evaluate the local environment. However, it is obtained from an invasive procedure with possible life-threatening complications. Instead, the exhaled breath condensate (EBC) collection is a non-invasive method for sampling airway-lining fluid, containing both volatile and nonvolatile compounds, and is currently accepted as a valuable tool for detecting markers of lung inflammation and oxidative stress [19]. 

Understanding the local immune and redox states would highly contribute to the biomarker field, and they could also be valuable for evaluating disease progression. Therefore, we designed this pilot study to determine the potential utility of unconcentrated EBC from adults with recently diagnosed pulmonary TB to evaluate inflammatory features, such as oxidative stress effectors, cytokines, and eicosanoids with a perspective on biomarkers discovery.

## 2. Materials and Methods

### 2.1. Study Group

We conducted a cross-sectional, prospective evaluation, designed as a pilot study to assess the potential utilization of the exhaled breath condensate (EBC) to evaluate immune-related biomarkers in patients with TB. The participants were recruited at the National Institute of Respiratory Diseases (INER) in Mexico City. The Institutional Ethics and Research Committee approved this study, and all participants provided written informed consent. We included patients recently diagnosed with pulmonary TB, who had not yet received anti-TB treatment, and healthy controls from both genders, aged between 18 and 65 years. Individuals with HIV infection, chronic inflammatory diseases, or cancer were excluded.

All patients were diagnosed with active TB after a sputum smear-positive test or Xpert^®^ MTB/RIF assay, further confirmed by M. tuberculosis culture. The subjects underwent radiological and clinical examinations. Disease severity was evaluated in terms of the smear report, laterality of the lung lesions, presence of cavities, and the score of radiographical abnormalities (SRA) as reported elsewhere [20]. The SRA was determined by assessing the presence, distribution, and extent of consolidation, fibrosis, lung distortion, bronchiectasis, and parenchymal abnormalities in four quadrants (axis spine and lower end of main bronchus in chest X-ray); each quadrant was scored from 0 to 5, where 0 indicated a normal appearance, and 5 indicated severe abnormality. The score represented the percentage of lung parenchyma involvement, and the maximum score was 20. The healthy controls had normal chest X-ray images and laboratory results. 

### 2.2. EBC Sample Collection

The EBC was collected per ATS/ERS recommendations [21]. Subjects breathed normally into an R-Tube device (Respiratory Research, Inc., Charlottesville, VA, USA), a disposable collection system that separates saliva from the exhaled breath, for 10 min. They did not eat, drink, or exercise for at least two hours before sample collection. No nose clips were used. All samples were taken in the morning, the room temperature was 22.7 °C (19.5–29.9), the humidity was 43% (20–73), and all participants had 12 h (2–14) of fasting. EBC samples were filtered through a 0.2 mm membrane, aliquoted, and immediately frozen at −80 °C until analysis. All samples were tested negative for salivary contamination by determination of α-amylase concentration (enzymatic colorimetric assay with a detection limit of 10 UI/L). The alveolar origin of the sample was confirmed by quantitation of the surfactant protein-A (SP-A) by ELISA using a Surfactant Associated Protein A kit (Cloud Clone Corp, Houston, TX, USA). The SP-A was present in all EBC samples (8.70, IQR 12.59 pg/mL for patients and 8.93, IQR 13.67 pg/mL for healthy controls). 

### 2.3. Serum Collection

Blood samples were obtained from the participants, and the serum was retrieved via centrifugation, aliquoted, and stored at −70 °C until use. The aliquots intended for the GSH determination were immediately mixed with a 1:1 volume of 0.6% sulfosalicylic acid and then centrifuged at 8000× *g* for 10 min at 4 °C. Next, the supernatants were transferred to a new tube and stored at −70 °C until use.

### 2.4. Redox State Biomarker Detection 

#### 2.4.1. Glutathione (GSH)

GSH was measured in serum using the GSH reductase enzyme method [22]. Briefly, the assay is based on the reaction of GSH (Sigma-Aldrich, St. Louis, MO, USA) with 5,5′-dithio-bis (2-nitrobenzoic acid) (DNTB, Sigma-Aldrich) to form 5-thio-2-nitrobenzoic acid (TNB), in the presence of β-NADPH (Sigma-Aldrich) and GSH reductase (Sigma-Aldrich). The absorbance at 412 nm was immediately measured at 30 s intervals for 2 min to compare with the GSH standard. The test is specific to GSH based on the specificity of the GSH reductase enzyme (Sigma-Aldrich) to GSH: the rate of accumulation of TNB is directly proportional to the concentration of GSH in the sample. 

#### 2.4.2. Malondialdehyde (MDA)

MDA is a naturally occurring product of lipid peroxidation used as a marker of cellular injury and an indicator of oxidative stress in cells and tissues. The Thiobarbituric Acid Reactive Substance (TBARS) is a method for monitoring lipid peroxidation. We detected MDA content in the EBS and serum using a TBARS Assay Kit (Cayman Chemical, Ann Harbour, MI, USA) according to the manufacturer’s instructions. The results are reported in μM of MDA. The assay range was 0.625–50 μM.

#### 2.4.3. 8-Isoprostane

The 8-isoprostane is an eicosanoid nonenzymatically derived through the free radical catalyzed metabolism of arachidonic acid. This compound is a biomarker of lipid peroxidation and indicates antioxidant deficiency and oxidative stress [23]. We measured this molecule in serum and EBC using an 8-isoprostane ELISA Kit (Cayman Chemical) according to the manufacturer’s instructions. The assay range was 0.8–500 pg/mL.

### 2.5. Nucleosomes’ Quantitation

Quantification of cell-free nucleosomes was performed using the Cell Death Detection ELISA Plus kit (Roche Diagnostics, Indianapolis, IN, USA), which detects DNA and histones (antibodies from the mouse clones M-CA-33 and H11–4) for the specific detection of mono- and oligonucleosomes, following the manufacturers’ instructions. Results are reported as a percentage of the positive control included in the kit as reported elsewhere [24].

### 2.6. Eicosanoids Detection

PGE2, LTB4, RvD1, and Mar1 concentrations were quantified in the EBC and serum using commercial EIA kits (Cayman). All assays were performed according to the manufacturer’s instructions. Serum samples underwent extraction using ethanol precipitation before analysis. Samples were analyzed in duplicates, and optical density was determined at 450 nm using a microplate reader (MultisKan Ascent). The PGE2 assay ranged from 15.6–2000 pg/mL and had a 36 pg/mL sensitivity. The LTB4 assay ranged from 3.9–500 pg/mL and had a 13 pg/mL sensitivity. The RvD1 assay ranged from 3.3–2000 pg/mL and had a 15 pg/mL sensitivity. The MaR1 assay ranged from 7.8–1000 pg/mL.

### 2.7. Cytokines Detection

Cytokines were measured in the EBC and serum samples using an 8-plex bioplex system (Bio-Rad, Hercules, CA, USA) for IL-2, IL-4, IL-6, IL-8, IL-10, IFN-γ, TNF-α, and GMC-SF according to the manufacturers’ instructions. Cytokine’s limit of detection, in pg/mL, were IL2 (1.6), IL4 (0.1), IL6 (0.5), IL8 (0.4), IL10 (0.3), TNFα (6.0), IFNγ (6.4) GM-CSF (2.2).

### 2.8. Statistics

Categorical data are presented as absolute numbers and percentages and continuous data as median and range. The Fisher’s exact test was used to compare categorical variables between study groups. Non-parametrical statistical methods were applied to continuous data. Significance of statistical difference was determined using the Kruskal-Wallis test with Dunn’s post-hoc for multiple comparisons, the Mann-Whitney U test for comparison of unpaired data, and the Wilcoxon rank test for matched pairs. Spearman’s Rho correlation was used to establish the correlation between variables. Analyses were performed using GraphPad Prism version 9.0 (GraphPad Software, La Jolla, CA, USA).

## 3. Results

### 3.1. Characteristics of the Study Group

We included twenty patients with pulmonary TB and twelve healthy subjects. Demographic and clinical variables are listed in Table 1. The mean age and sex of the TB patients were similar to the healthy controls. The average body mass index (BMI) in the TB group was significantly lower than in the healthy controls, reflecting the catabolic nature of TB disease. Among patients with TB, the median age was 47.5 years old (range 18–64), there were 13 males (65%), and thirteen patients (65%) had diabetes, two hypertension, and two smoking histories. Most of the patients presented bilateral lesions (70%) with sputum smear grade 1+ (50%), and the median score of radiographical abnormalities was 10 (range 2–18). Patients had high WBC, neutrophil, lymphocyte, and monocyte peripheral blood counts (Table 1). Regarding the EBC collection, samples were obtained at similar environmental conditions. However, more EBC volume was collected from the healthy controls (Table 2).

### 3.2. Redox Exhaled and Systemic Markers

**Figure 1:**
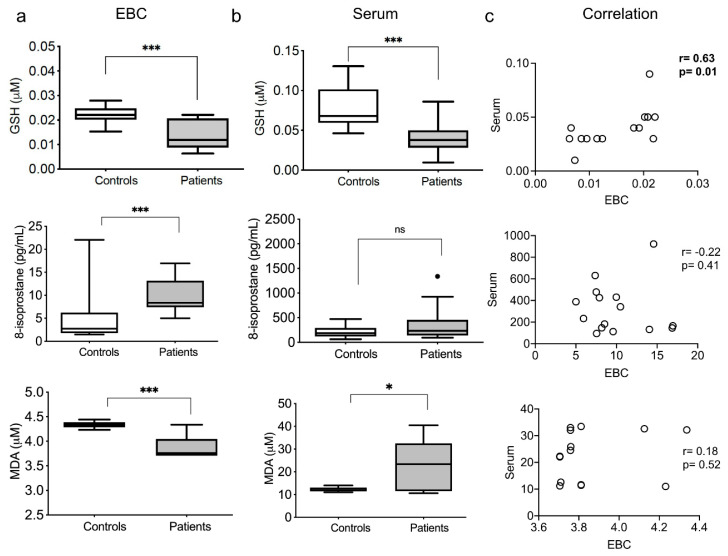
Elevated exhaled and systemic levels of oxidation markers. The levels of GSH, 8-isoprostane, and malonaldehyde (MDA) were measured in exhaled breath condensates (EBC, **a**) and serum (**b**) of patients and healthy controls. Spearman’s correlation was calculated for the patients’ data (**c**). Box plots depict medians and quartiles; correlation plots depict individual results, Spearman’s rho, and *p* values. * *p* < 0.05, *** *p* < 0.001, ns = non-significant.

GSH (reduced glutathione) is a potent antioxidant involved in maintaining and regulating the thiol-redox status. EBCs and sera from healthy subjects showed significantly higher concentrations of GSH than TB patients. In the same way, the serum of healthy controls presented a higher concentration compared to TB patients (Figure 1a,b, upper panel).

The 8-isoprostane is considered a reliable biomarker of lipoperoxidation, antioxidant deficiency, and oxidative stress [25]. EBCs from TB patients showed a significantly increased concentration of 8-isoprostane compared to healthy controls. In the same way, the sera of TB patients presented a higher 8-isoprostane concentration, although not significant (Figure 1a,b, middle panel). 

MDA is a dialdehyde, and highly reactive hydroperoxide, and the more stable lipoperoxidation product. Surprisingly, the EBCs from healthy subjects showed higher concentrations of MDA than TB patients. In contrast, the serum from healthy volunteers presented a reduced MDA concentration compared to TB patients (Figure 1a,b, lower panel).

Comparing the redox state markers patterns between alveolar and systemic samples, we found a weak positive correlation between the EBC and serum GSH levels from TB patients (Figure 1c, upper panel). We did not observe a correlation between EBC and serum 8-isoprostane and MDA levels in the patients (Figure 1c, middle and lower panels). Also, we observed no correlation between EBC and serum for the three redox parameters in the healthy controls (Supplementary Figure S1).

### 3.3. Exhaled and Systemic Cytokines

**Figure 2:**
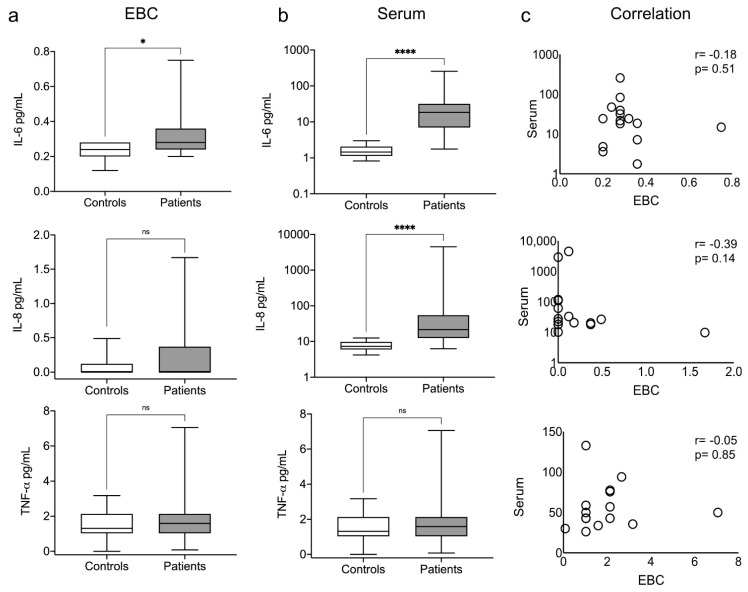
Elevated exhaled and systemic levels of pro-inflammatory cytokines. IL-6, IL-8, and TNF-α levels were measured in the EBC (**a**) and serum (**b**) of patients and healthy controls. Spearman’s correlation was calculated for the patients’ data (**c**). Box plots depict medians and quartiles; correlation plots depict individual results, Spearman’s rho, and *p* values. * *p* < 0.05, **** *p* < 0.0001, ns = non-significant.

We surmised an elevation in the concentration of the pro-inflammatory cytokines IL-6, IL-8, and TNF-α in the EBC and serum of patients. However, we observed that only IL-6 was significantly elevated in the EBC of the patients, whereas IL-6 and IL-8 were significantly elevated in the serum (Figure 2a,b, upper and middle panels). We did not find TNF-α elevated in any study groups or samples (Figure 2a,b, lower panel). We found no differences in the other cytokine levels between the study groups or samples (Supplementary Figure S2a). Despite the elevated levels of IL-6 in both samples, there was no correlation between the samples in any case (Figure 2c, and Supplementary Figure S2b).

### 3.4. Exhaled and Systemic Nucleosomes

**Figure 3:**
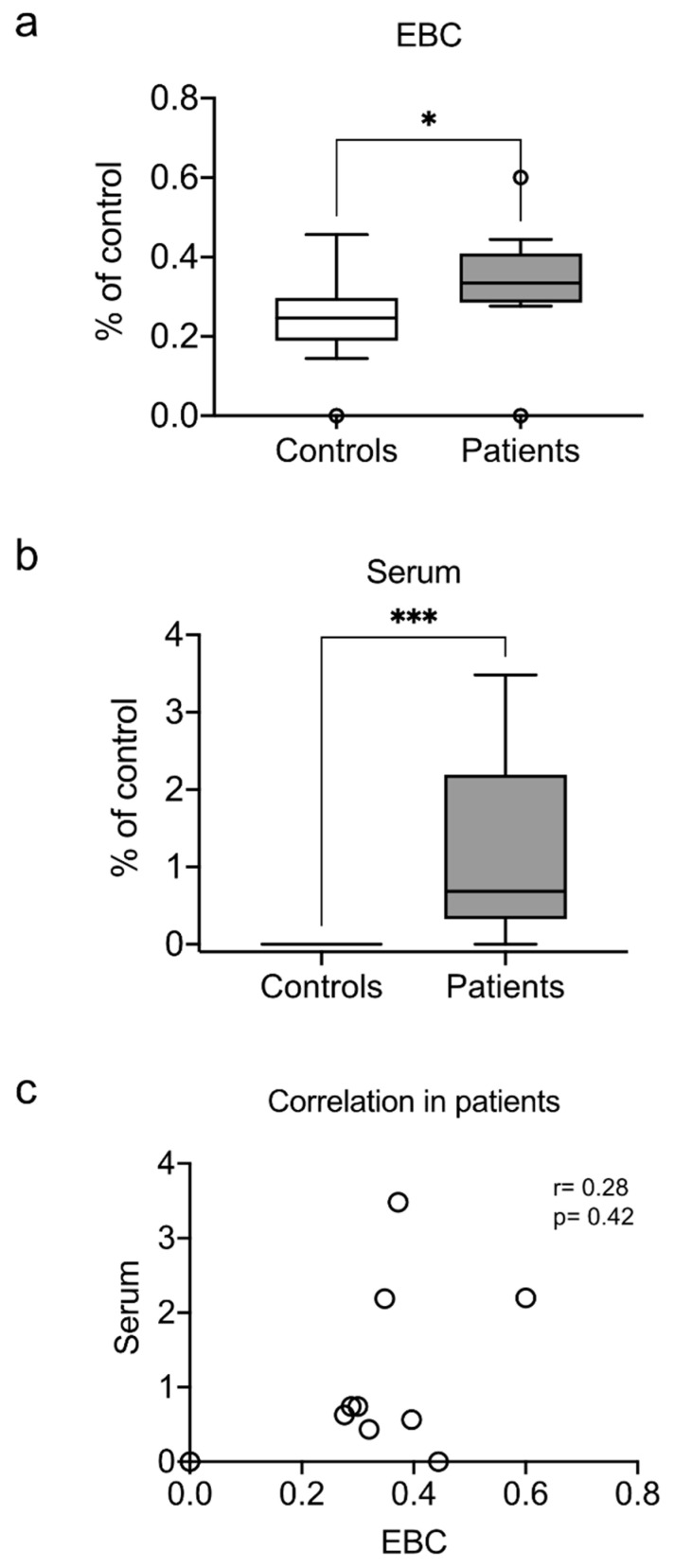
Elevated exhaled and systemic nucleosomes. The levels of cell-free nucleosomes were measured in the EBC (**a**) and serum (**b**) of patients and healthy controls. Spearman’s correlation was calculated for the patients’ data (**c**). Box plots depict medians and quartiles; correlation plots depict individual results, Spearman’s rho, and *p* values. * *p* < 0.05, *** *p* < 0.001.

Cell-free nucleosomes are released during necrosis and neutrophil degranulation and are frequently used as surrogates of neutrophil extracellular traps (NETs) release [26]. NETs associate with compromised lung function during pneumonia due to alveolar damage and epithelial cell death [27]. Nucleosomes are a marker of pulmonary damage and excess inflammation in patients with chronic obstructive pulmonary disease (COPD) [26,28]. Elevated circulating nucleosomes have also been reported in TB [29]. Therefore, we investigated whether cell-free nucleosomes can also be found in EBC. We found elevated levels of nucleosomes in the EBC from patients (Figure 3a). As expected, we also observed elevated levels of nucleosomes in the serum (Figure 3b). However, despite being elevated in both samples, there was no correlation between EBC and serum nucleosome levels (Figure 3c).

### 3.5. Exhaled and Systemic Eicosanoids

**Figure 4:**
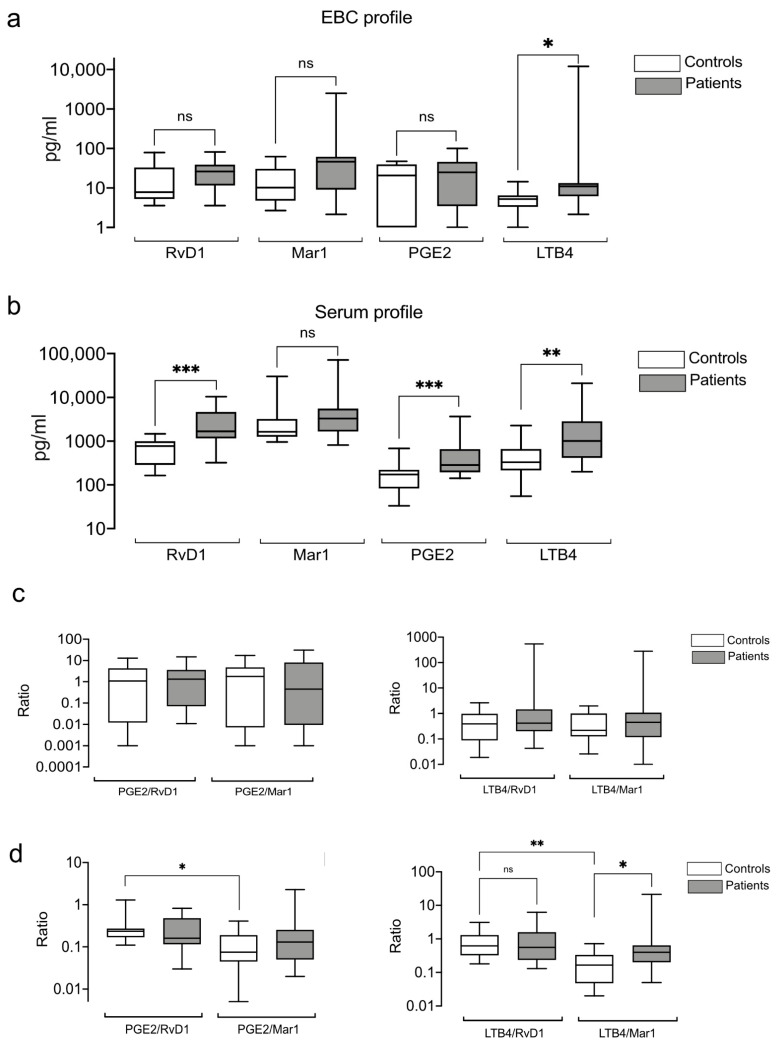
Exhaled and systemic eicosanoids profiles. Pro-resolving mediators RvD1 and Mar1 and pro-inflammatory PGE2 and LTB4 were measured in the EBC and serum of patients and healthy controls. The alveolar (**a**) and systemic (**b**) profile was analyzed for individual levels of lipids concentrations. The relationship between pro-inflammatory and pro-resolving lipid mediators was calculated as PGE2/RvD1, PGE2/Mar1, LTB5/RvD1, and LTB4/Mar1 ratios in EBC (**c**) and serum (**d**). Box plots depict medians and quartiles. * *p* < 0.05, ** *p* < 0.01, *** *p* < 0.001, ns = non-significant.

We analyzed the PGE2, LTB4, RvD1, and Mar1 levels and observed similar profiles in EBC and serum for patients and healthy controls (Supplementary Figure S3). We also found no significant differences in RvD1, Mar1, and PGE2 between the groups in the alveolar samples (Figure 4a). However, the levels of LTB4 were significantly elevated in the EBC of the patients (Figure 4a). In contrast, serum concentrations of PGE2, RvD1, and LTB4 were higher in patients than in healthy controls (Figure 4b). There was no correlation between EBC and serum lipid levels in patients and controls (Supplementary Figure S4). Because eicosanoid effects may depend on their relative contribution rather than absolute levels [17], we investigated the following pro-inflammatory/pro-resolving eicosanoid ratios: PGE2/RvD1, PGE2/Mar, LTB4/RvD1, and LTB4/Mar1. We found that none of the eicosanoid ratios in EBC distinguished the patients from the healthy subjects (Figure 4c). LTB4/RvD1 and LTB4/Mar1 were higher in the EBCs of the patients, but they did not reach significance. However, the serum eicosanoid relationships revealed a significant difference between the PGE/RvD1 and PGE2/Mar1 and between LTB4/RvD1 and LTB4/Mar1 in the healthy subjects (Figure 4d). Besides, the LTB4/Mar1-ratio distinguished the patients from the healthy controls.

### 3.6. Redox and Pro-Inflammatory Markers Correlation

**Figure 5:**
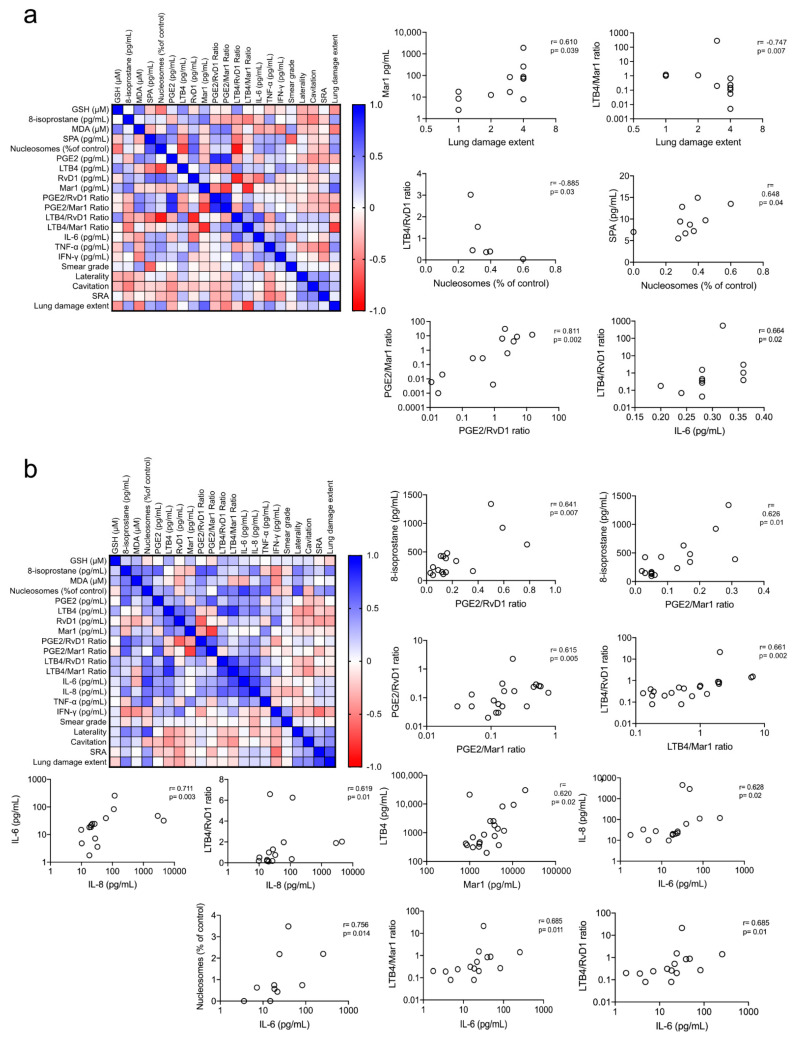
Correlation among redox, eicosanoids, and cytokines biomarkers, and the severity of the disease. We performed a correlation matrix including all the metabolites in the study to evaluate whether the alveolar (**a**) and systemic (**b**) biomarkers correlated with one another. The correlation plots of the pairs with correlation >0.6 or <−0.6 with a *p*-value < 0.05 are depicted, excepting the obvious correlation of PGE2, LTB4, RvD1, and Mar1 with their corresponding ratios. Correlation plots depict individual results, Spearman’s rho, and *p* values.

Because we found several of the three biomarkers (redox, eicosanoids, and cytokines) significantly elevated in the EBCs from TB patients, we investigated if they were associated with any of the evaluated features. Because the homogeneity of the study group prevented their categorization into subgroups, we performed a correlation matrix including all the metabolites in the study to evaluate whether any of them exhibited a correlation among one another (Figure 5a,b). We found a significant correlation among several parameters of the three systems of biomarkers in the study. We selected for display the ones with correlation >0.6 or <−0.6 with a *p*-value < 0.05, excepting the obvious correlation of PGE2, LTB4, RvD1, and Mar1 with their corresponding ratios. We observed no correlation between the metabolites in the alveolar sample and the smear grade (Figure 5a). However, we found that lung damage extent, measured as the number of quadrants with radiographic abnormalities, showed a positive correlation with Mar 1 and a negative correlation with LBT4/Mar1ratio. Furthermore, LTB4/RvD1 ratio positively correlated with IL-6 levels and negatively correlated with GSH levels linking the three aspects of redox and inflammation markers in the lungs. Also, SPA positively correlated with the level of nucleosomes, and LTB4/RvD1 ratio negatively correlated with the level of nucleosomes (Figure 5a). 

When analyzing the serum level of the metabolites, we observed no correlation of any of them with the smear grade or lung damage extent (Figure 5b). Nonetheless, the 8-isoprostane levels correlated with the PGE2/RvD1 and PGE2/Mar1 ratios linking the redox with the eicosanoid markers; also, PGE2/RVD1 and PGE2/Mar1 ratios correlated with LTB4/RvD1 and LTB4/Mar1 ratios. Interestingly, the PGE2/RvD1 ratio correlated with the levels of IL-6 like the one observed in the alveolar sample (Figure 5b).

## 4. Discussion

Changes and dynamics in peripheral blood may not reflect the local immune responses to *M. tuberculosis* infection. In this pilot study, we evaluated critical components of severe inflammation: oxidant/antioxidant elements, pro-inflammatory cytokines, cell-free nucleosomes, and eicosanoids in EBC from patients recently diagnosed with pulmonary TB. We contrasted the results with the serum levels of the selected biomarkers.

Oxidative stress is provoked by exacerbating ROS production through the respiratory burst and reducing the antioxidant effectors [30,31]. This condition affects normal lung function and the host immune responses contributing to disease pathogenesis due to the inability to remove the oxidative burden properly, ultimately generating pulmonary dysfunction [7,31]. We observed reduced levels of the antioxidant GSH in EBC of patients, indicating alveolar oxidative stress that has not been reported before. We also found this GSH reduction in the serum. The reduction in the circulating levels of antioxidant molecules such as GSH and others has been reported in TB [7,31,32]. 

Circulating antioxidant effectors are insufficient to counteract the oxidative stress in TB, and free radicals presumably accumulate within pulmonary tissue attacking membrane lipids, leading to lipid peroxidation [30,31]. Here, we confirm a deficiency in the antioxidant system at the alveolar level in TB. Also, the high levels of MDA in the serum of patients suggest increased lipid peroxidation rates and dysfunction of redox homeostasis [33]. Unexpectedly, MDA was significantly low in the EBC of patients. In contrast, the 8-isoprostane was significantly elevated in the EBC of patients. However, it was not different in the serum between groups, suggesting that the 8-isoprostane is a local oxidative marker that reflects the altered oxidizing environment in the airways of patients with TB, contributing to progressive lung disease. The 8-isoprostane is a prostaglandin that increases in an oxidative condition such as hypoxia and can activate phosphatidic acid A2 to hydrolyze phospholipids on the cell membrane, then releasing arachidonic acid and then activating phospholipase C, which can decompose inositol triphosphate and rerelease arachidonic acid, thus increasing the isoprostanes level [34]. 

When evaluating the inflammatory markers, we found higher concentrations of IL-6 in the EBCs and sera from TB patients than in healthy controls. This cytokine is induced and required to prompt an initial protective IFN-γ response during *M. tuberculosis* infection. This early response occurs in the lung and is essential for the initial control of mycobacterial growth [35]. We detected high systemic levels of IL-8 in TB patients, but it was probably associated with the sample type because IL-8 is excessively high in cerebrospinal fluid, bronchoalveolar lavage fluid, and tuberculous pleural exudate suggesting its association with damage [36,37]. Additionally, some studies have shown higher plasma levels of IL-8 in patients who died from TB then the levels in survivors [38]. Furthermore, the generation and release of IL-8 by leukocytes have been described in response to *M. tuberculosis* or its components [39]. Despite not reaching significance, the slightly elevated levels of lL-8 we found in unconcentrated EBCs confirmed inflammation and possible damage at the site of infection. 

After infection, TNF-α is abundant and is released in the lung of TB patients, indicating active cytokine stimulation. Various cell types are endowed with the propensity to produce TNF-α, yet mononuclear phagocytes represent the dominant cellular source of this cytokine. The production of proinflammatory cytokines can reach the systemic circulation where they may cause systemic symptoms [40]. Cytokine concentrations principally determine the duality of TNF-α in conferring protection or pathology, yet interference with additional signaling pathways deserves consideration. Adequate TNF-α amounts confer adequate responses limiting bacterial burdens, while excessive levels induce pathology through hyperinflammation [41]. 

Although TNF-α, IFN-γ, and other cytokines play important roles in the lung environment during TB infection, we did not observe significant levels of any of them in the patients’ EBC. It was unlikely related to the stage of the disease because high systemic levels of IL8 were detected in the patients, but it was probably associated with the source of sampling. We cannot determine if these proteins are quickly exhaled, but the protein content of the EBCs was sufficient to allow the evaluation of SPA and the nucleosomes. Consequently, cytokines may not be good markers to be evaluated in EBC.

Thus, we looked for other markers associated with inflammation. Neutrophils release nucleosomes as a host antimicrobial defense mechanism typically associated with compromised lung function due to cell death and tissue damage [26,29]. The increase of nucleosome levels in plasma and sputum samples from patients with TB has been reported, and their baseline levels correlated with disease severity and decreased with antibiotic therapy [29,42]. Here, we found higher nucleosome levels in EBC and serum, suggesting that their release occurs in the lungs and reaches systemic circulation revealing chronic inflammation and tissue damage in the patients.

In addition, we detected high levels of LTB4 in the EBC of the patients. LTB4 is a pro-inflammatory lipid mediator that acts as a chemoattractant of neutrophils, and it may be released by macrophages, epithelial cells, and activated neutrophils [43]. The increase of LTB4 has been reported in plasma from TB patients [44]. LBT4 is considered an inflammatory marker in EBC from patients with chronic obstructive pulmonary disease [45]. Here, we also provide evidence that LTB4 may be an alveolar biomarker in TB.

Finally, we performed a correlation matrix to establish the relationship among the measurements. The serum results confirmed a systemic inflammatory condition in patients with TB because the inflammatory markers correlated. However, it did not reflect the degree of lung damage. In contrast, the inflammatory mediators such as LTB4, Mar1, and the cell-free nucleosomes in EBC correlated among them and the radiological parameters of lung damage. We found no associations with other parameters of the disease severity, such as smear grade or cavities. 

## 5. Conclusions

In summary, this pilot study provided evidence that we can use the EBC to study alveolar biomarkers associated with lung damage, such as the release of nucleosomes, 8-isoprostane, GHS, IL-6, and LTB4. The lack of correlation between EBC and serum highlights the importance of measurement of local metabolites. These biomarkers have the potential to evaluate disease progression and lung damage, provide pathophysiologic insight before and after the initiation of conventional therapy to assess treatment success, and aid in the design of new therapy approaches that include the use of antioxidants.

## Figures and Tables

**Table 1:** Characteristics of the study participants.

	TB Patients (N = 20) Total (%) or Median (Range)	Healthy Controls (N = 12) Total (%) or Median (Range)	*p* Value
*Demographics*			
Age, year	47.5 (18–64)	50 (31–62)	0.751
Sex, male (%)	13 (65)	8 (66.6)	0.999
BMI	21.8 (12.7–39.7)	23.8 (21.3–27.5)	0.078
*Comorbidities*			
Diabetes (%)	13 (65)	0 (0)	0.999
Hypertension (%)	2 (10)	0 (0)	0.999
Smoking (%)	2 (10)	1 (8.3)	0.999
*Tuberculosis*			
Unilateral	6 (30)	-	
Bilateral	14 (70)	-	
*Smear grade*			
0+	4 (20)	-	
1+	10 (50)	-	
2+	1 (5)	-	
3+	5 (25)	-	
Score of radiographical abnormalities *	10 (2–18)	-	
*Leukocytes*			
WBC count, ×10^9^/L	8.6 (4.1–15.2)	6.6 (3.6–8)	<0.001
Neutrophil count, ×10^9^/L	6.3 (2.2–13)	3.8 (1.2–4.3)	<0.001
Lymphocyte count, ×10^9^/L	1.1 (0.5–2.4)	2.1 (1.6–2.8)	<0.001
Monocyte count, ×10^9^/L	0.8 (0.1–1.5)	0.5 (0.3–0.7)	<0.001
Platelet count, ×10^9^/L	335 (13.3–662)	252 (205–373)	0.122

* The score represented the percentage of lung parenchyma involvement. The maximum score was 20, as stated in the methods section. WBC = white blood cell.

**Table 2:** EBC collection data.

	TB Patients (N = 20)	Healthy Controls (N = 12)	*p* Value
Fasting, h	12 (2–14)	12 (10–16)	0.540
Total volume, L/10 min	1050 (260–1600)	1350 (480–2300)	0.041
Room temperature, °C	22.7 (19.5–29.9)	22.5 (21.3–22.8)	0.263
Humidity, %	43 (20–73)	43.5 (35–50)	0.726

## Data Availability

The data used to support the findings of this study are included within the article.

## Supplementary Materials

The following are available online at https://www.mdpi.com/article/10.3390/antiox10101572/s1, Figure S1: Correlations between alveolar and systemic levels of oxidation markers in healthy controls., Figure S2: Alveolar and systemic cytokines., Figure S3: Comparison of individual expression of alveolar and systemic eicosanoids., Figure S4: Correlations between alveolar and systemic levels of eicosanoids.
