# Supplementary material for: Oxidative Stress and Inflammatory Mediators in Exhaled Breath Condensate of Patients with Pulmonary Tuberculosis. A Pilot Study with a Biomarker Perspective
Source: Antioxidants (Basel). 2021 Oct 5;10(10):1572. doi: 10.3390/antiox10101572 (PMC8533495; doi:10.3390/antiox10101572)
Supplement: Supplementary file 1 [file antioxidants-10-01572-s001.zip › antioxidants-1358473-supplementary.pdf]

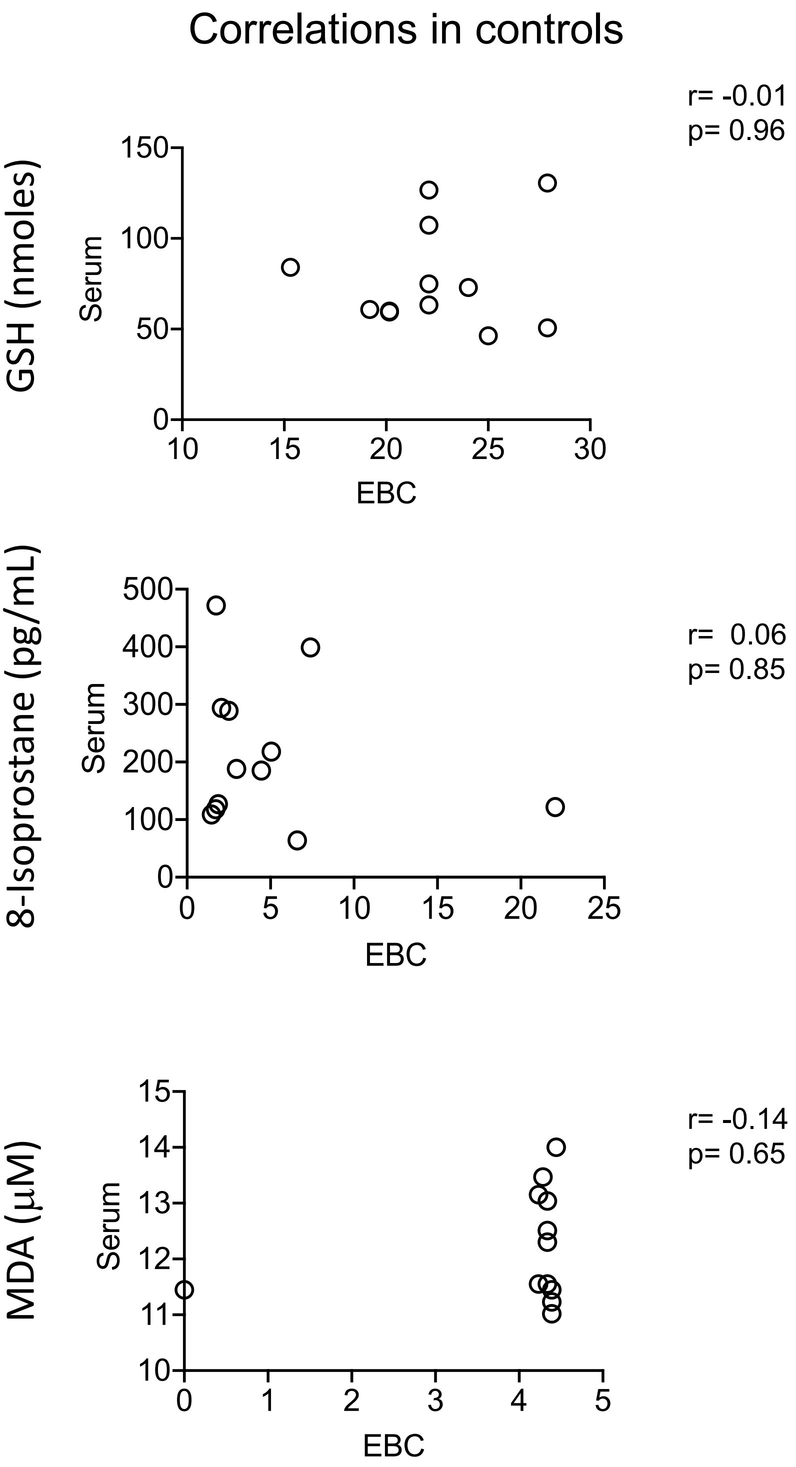

Supplementary Figure S1. Correlations between alveolar and systemic levels of oxidation markers in healthy controls. Levels of GSH, 8-isoprostane, and malonaldehyde (MDA) were measured in exhaled breath condensates (EBC) and sera from healthy controls. Correlation plots depict individual results, Spearman's rho, and *P* values.

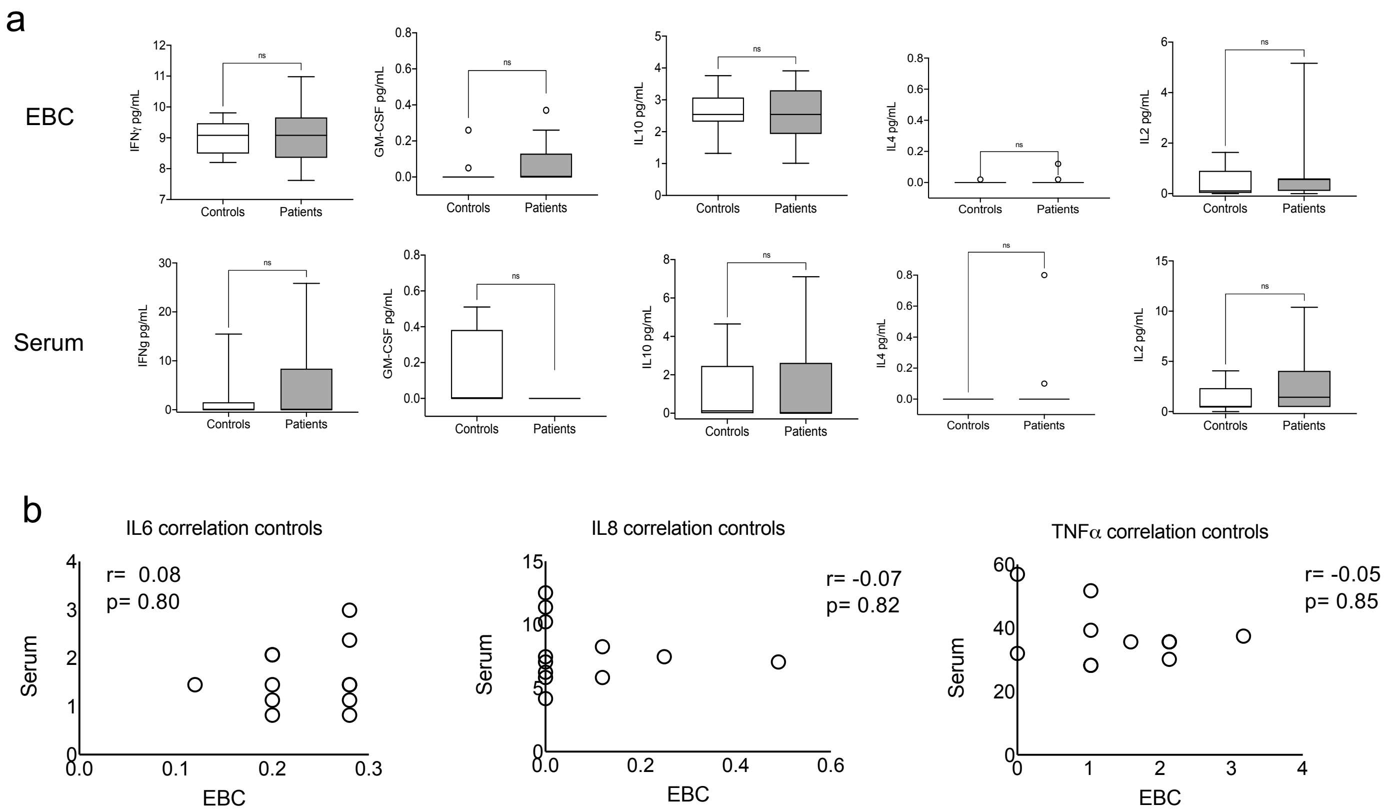

Supplementary Figure S2. Alveolar and systemic cytokines. The levels of IFN-γ, GM-CSF, IL-10, IL-4, and IL-2 were measured in the EBC and sera of TB patients and healthy controls (a). Spearman's correlation was calculated for the healthy control data depicted in Fig. 2, IL-6, IL-8, and TNF-α data (b). Box plots depict medians and quartiles; correlation plots depict individual results, Spearman's rho and *P* values.

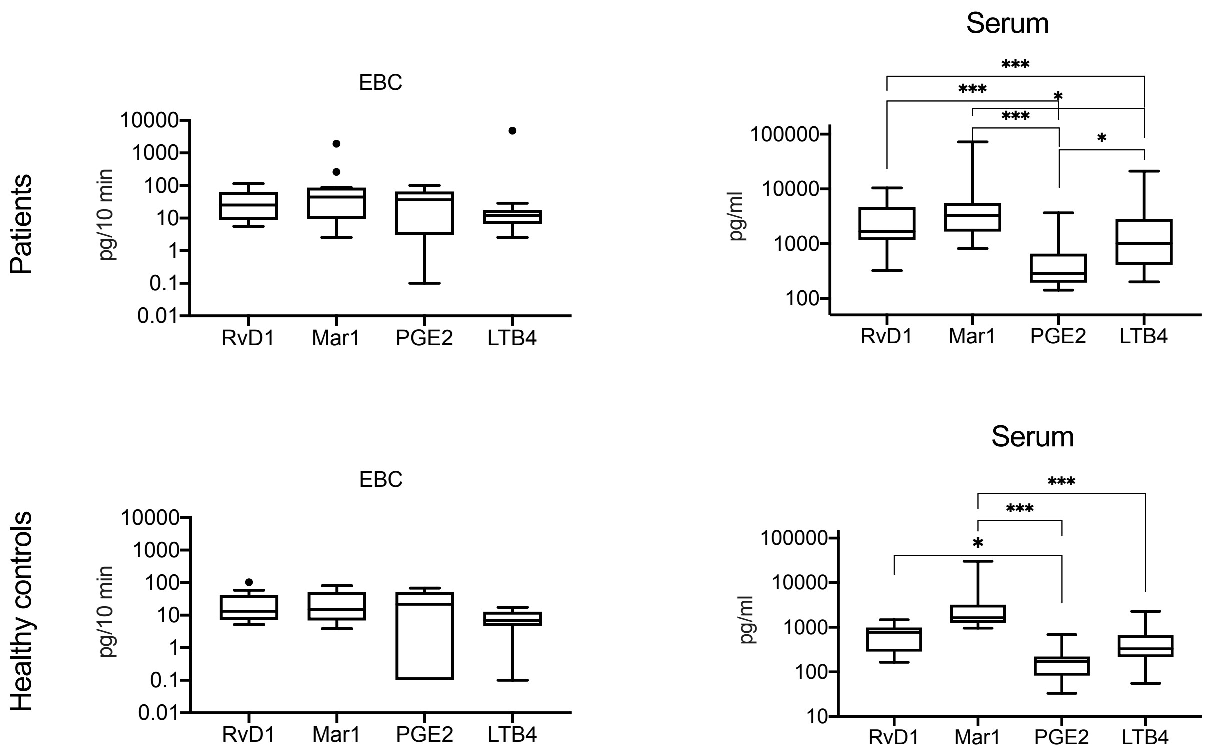

Supplementary Figure S3. Comparison of individual expression of alveolar and systemic eicosanoids. Levels of the pro-resolving mediators RvD1 and Mar1, and the pro-inflammatory PGE2 and LTB4 were measured in the EBC and serum of TB patients and healthy controls. *P<0.05, ***P<0.001.

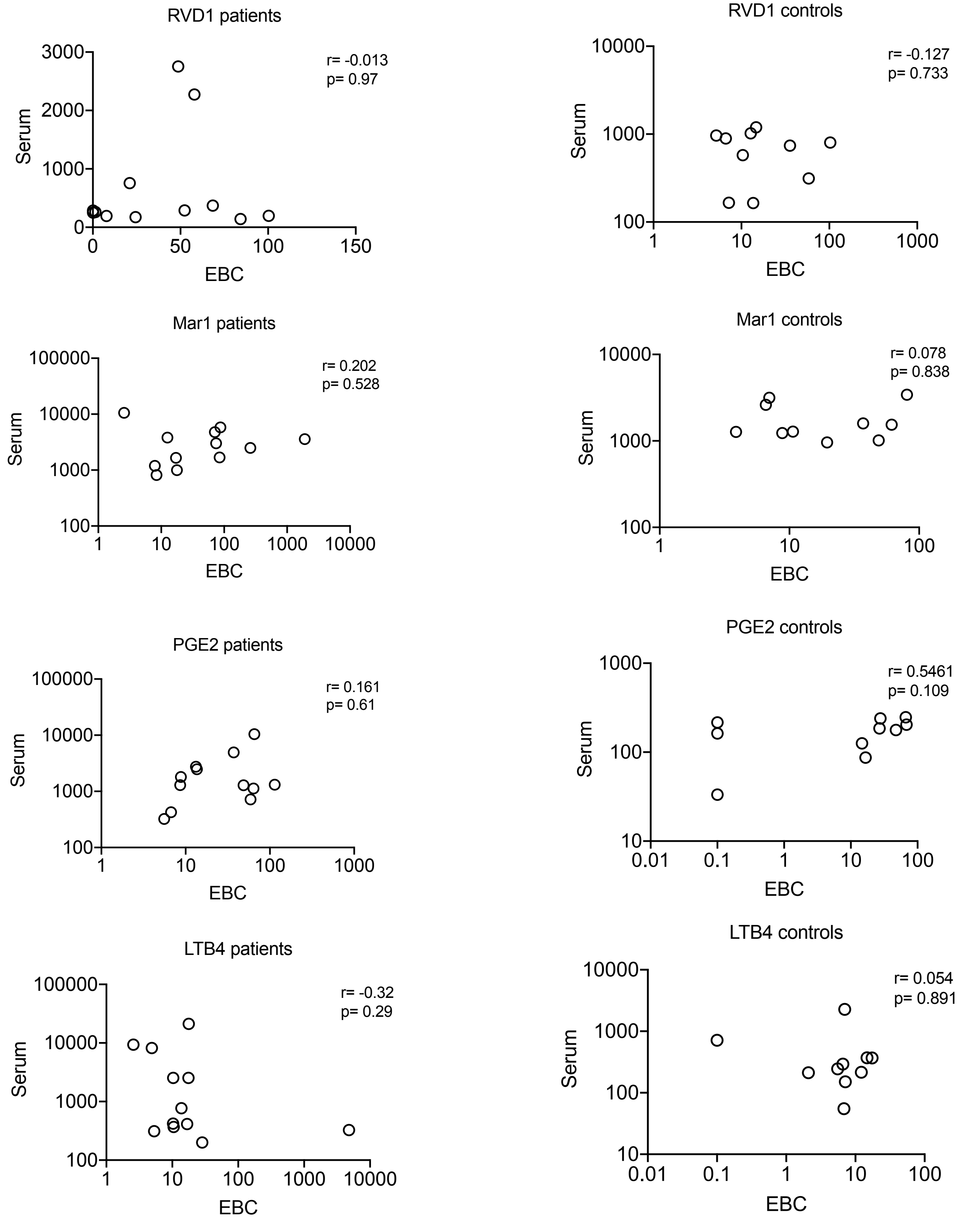

Supplementary Figure S4. Correlations between alveolar and systemic levels of eicosanoids. Levels of the pro-resolving mediators RvD1 and Mar1, and the pro-inflammatory PGE2 and LTB4 were measured in EBC and serum. Spearman's correlation was calculated for the patients' data (left) and healthy controls (right). Correlation plots depict individual results, Spearman's rho, and *P* values.
